# Comments and reflections on ITS and STEM education and training

**DOI:** 10.1186/s40594-018-0106-7

**Published:** 2018-04-17

**Authors:** J. D. Fletcher

**Affiliations:** Science and Technology Division, Institute for Defense Analyses, 4850 Mark Center Drive, Alexandria, VA 22311 USA

**Keywords:** Individualization in learning, Intelligent tutoring systems, Instructional objectives and standards, Natural language dialogue, Mixed initiative dialogue

## Abstract

This article provides brief comments and reflections on Intelligent Tutoring Systems (ITS) and their use in providing STEM (science, technology, engineering, and mathematics) education and training as described in the four articles prepared for this special issue concerning the ONR (Office of Naval Research) STEM Grand Challenge. General points raised include the need for individualization in education and training and the need for STEM instruction in all sectors of the economy, especially the extensive education and training requirements of national defense. Other comments concern the role of ITS as an instructional approach in providing STEM education and training in general and in comparison with other computer-assisted approaches. Additionally, they discuss the establishment of STEM objectives and standards for ITS, with its promise to accelerate acquisition of technical expertise, and the use of mixed initiative, natural language dialogue to provide tutorial direction, advice, and hints.

## Background

These comments begin with brief background discussions about the general need for individualization and tutorial instruction, the specific need for education and training in STEM (science, technology, engineering, and mathematics) topics, and the role and potential of Intelligent Tutoring Systems (ITS) in providing it. They then turn to more specific comments and reflections arising from the articles prepared for this special issue on the application of ITS in STEM instruction.

## Individualization in education and training

In 1906, E.L. Thorndike, noted that: “the principal consequence of individual differences is that every general law of teaching has to be applied with consideration of the particular person [because] responses to any stimulus will vary with individual capacities, interests, and previous experience” (page 83). His observation was supported by Gettinger and White (1980) whose research found that the rate with which students in a typical elementary school classroom learn differs by a factor of about 4:1. This ratio has been found by others. For instance, data from Suppes et al. (1975) show the same ratio in learning rate trajectories of students in grades 4–5.

These findings suggest that an appreciable number of students in any classroom will be struggling to learn while others are waiting and losing time to develop their potential. It also emphasizes the importance of individualization in education and training. Because learning rates depend to a large degree on prior knowledge (e.g., Tobias 2003, among others), the need for individualization also increases with age as the amount and diversity of learners’ experience increases.

It is difficult to deny the economic and social advantages of classroom instruction, but it is not an optimal provider of learning. Bloom’s research and that of his students (1984) indicated a learning increase of two standard deviations in comparison of tutoring (one instructor working with one learner) with classroom instruction. This increase is (roughly and on average) equivalent to raising the scores of 50th percentile learners to the 98th percentile. Discussion continues about these findings, but an effect size of 2 standard deviations remains both a target for research and development and an indicator of the value of individual tutoring over classroom instruction—despite heroic attempts by classroom teachers to overcome its limitations.

Learning may be substantially increased by providing one human tutor for each learner—an approach that is economically infeasible for all but a limited number of critical and demanding subjects such as surgery and aircraft piloting. As a practical matter, then we cannot afford a human tutor for every learner. However, we can afford computer access, if not a computer itself, for every learner, and computer-based tutoring can provide education and training with “consideration of the particular person [that] will vary with individual capacities, interests, and previous experience”. With that, we come to digital tutoring, or intelligent tutoring systems (ITS), which are intended to provide tutorial instruction for each learner.

The Department of Defense has long been a leader in the development of ITS (Fletcher 2009). It must annually prepare thousands of individuals ab-initio (assuming no prior knowledge or experience) to perform highly technical tasks and occupations. This preparation must be done as efficiently and reliably as possible in enabling the military services to perform their missions. Promising training technologies, from simulation to ITS, are therefore a priority for military research and development.

The Office of Naval Research (ONR) is notable for its long-standing support of all levels of computer-assisted education and training. It provided funding for research reported by the first books in this area (Crowder 1959; Coulson 1962) and, soon thereafter, design and development of the MENTOR programming language and the MENTOR instructional program (Feurzeig 1969), which evidently was the first ITS (a term that emerged later). MENTOR was based on the insight by Uttal (1962), Swets and Feurzeig (1965), and other early researchers that computers could do more than simply provide automated workbooks or programmed learning texts. MENTOR was intended to engage in tutorial conversations that would train students, ranging from independent duty corpsmen to novice physicians, to perform medical examinations—a typical STEM issue. Today, ONR is continuing its support of ITS and, among other matters, ITS applied to STEM education and training at all levels of learning.

## STEM

STEM focuses on science, technology, engineering, and mathematics, but these disciplines are broadly defined in STEM practice. The Bureau of Labor Statistics classifies 97 occupations as STEM (Cover, Jones, and Watson 2011). The National Science Foundation classifies 122 education degree fields as STEM, with continuing discussion about what qualifies as STEM, STEM occupations, and STEM skills (Department of Defense 2015). However identified, there is a steadily increasing need to prepare all levels of the national workforce with STEM knowledge and skills (National Academies of Sciences, Engineering, and Medicine 2017).

In 2013, the Committee on Science, Technology, Engineering, and Math Education (CoSTEM), which was assembled for the White House Office of Science and Technology, published a STEM education 5-year strategic plan (*Federal Science, Technology, Engineering, and Mathematics (STEM) Education 5-Year Strategic Plan* 2013) while noting that:

- Average mathematics and science literacy scores in the USA are below those for all other developed countries.
- The USA has fewer high scores and more low scores than those in other countries.
- Although STEM proficiency is essential for scientists and engineers who work in research and development (R&D), STEM knowledge and habits of mind are also required by workers in non-R&D occupations to perform a wide variety of activities.
- Although many individuals with STEM degrees do not work in STEM fields, they report that their STEM education is relevant, if not essential, in performing their job assignments.
- Even without formal degrees, possession of STEM abilities open up opportunities for individuals in both STEM and non-STEM occupations.

Carnevale and Smith (2013) and Donachie (2017) identified basic cognitive capabilities that are of value within and beyond STEM disciplines and education. These capabilities include critical thinking, communication, collaboration, deductive and inductive reasoning, and other habits of mind that are emphasized in STEM education and that support the ability to perform investigative and independent work in any area. They are particularly targeted and well supported by ITS systems, which have been especially effective in developing them.

In sum, individuals at all levels of activity now need STEM skills as well as STEM knowledge to function effectively in a modern economy. Helping them acquire STEM skills and knowledge is essential—a worthy and appropriate objective for ITS.

## ITS and STEM

Most applications of ITS appear to concern STEM education and training. For instance, of the 50 ITS assessments in a recent meta-analysis by Kulik and Fletcher (2016), all but 3 focused on STEM subject matter. One likely reason for this emphasis is that ITS developers are themselves scientists, technologists, engineers, and mathematicians. But another reason may be that ITS meet specific and increasing needs to prepare individuals to perform technical tasks and occupations in all sectors of the economy.

The four central articles in this issue of the International Journal of STEM Education are introduced by Craig et al. (2018). The articles concern research on ITS features and designs for STEM education and training. This research itself involves a full range of memory, cognition, learning, and behavior, all well within STEM territory. Nye et al. (2018) describe the use and effectiveness of natural language tutorials in algebra. Inventado et al. (2018) discuss the design and use of hints and other help activities to provide instruction in middle school mathematics. Skinner et al. (2018) describe the extension of task analysis needed for STEM activities into perceptual and psychomotor requirements for surgeons training to perform Robotic Assisted Laparoscopic Surgery (RALS). Finally, Graesser et al. (2018) discuss the integration of lessons learned from integrating five ITS systems to provide post-secondary school instruction in electronic circuits.

Comments here concern four issues underlying the design and use of ITS in STEM education suggested by the articles contributed to this issue. These comments concern the general role of ITS education and training, need for explicit objectives and standards in STEM ITS, the opportunity to accelerate development of STEM expertise, and the use and need for mixed initiative dialogue in ITS.

## The role of ITS in STEM education and training

Articles in this issue concern academic disciplines beginning at the middle school level and upward. Of the 50 ITS studies that met criteria for meta-analysis selection by Kulik and Fletcher (2017), only 2 were intended for learners below grade 6. Earlier education levels focus more on nomenclature, procedures, and processes such as 2-column addition, spelling patterns for reading, and the names of capital cities. Introductory instruction in any discipline and at any level is likely to involve similarly elementary matters. The value of ITS appears to increase along with the need to deal with the complexity, multi-level interactions, and abstract concepts that are required for higher levels of knowledge and technical skill. For instance, Kulik and Fletcher found that effect sizes of ITS in elementary and secondary instruction averaged 0.44 compared to 0.75 for post-secondary instruction.

Nye et al. (2018) and Graesser et al. (2018) note the effort and expertise needed to design and develop ITS. The greater reliance on ITS to present abstract, construct-driven material may reflect and suggest pragmatic judgments about the learning objectives for which applications of ITS are most likely to provide greater return on investment. This point is not to suggest that ITS cannot or should not be used at earlier grade levels or to provide the rudimentary subject matter. It is more concerned with the cost-effectiveness and return on investment from ITS given the cost and effort now needed to design and develop ITS. The Generalized Intelligent Framework for Tutoring (GIFT) (Sottilare et al. 2012) and other initiatives to support ITS development along with efforts to enable computers themselves to assume most of this labor—on demand and in real time (e.g., Dodds and Fletcher 2004; Fletcher 2009)—may soon further reduce costs and increase return on investment sufficiently to motivate their wider use. Cases are appearing that report very large savings and return on investment from ITS applications that produce essential competencies, thereby replacing the years of experience and on-the-job training that would otherwise be needed (Cohn and Fletcher 2010; Fletcher 2017).

## Pairing ITS with drill and practice

**Fig. 1:**
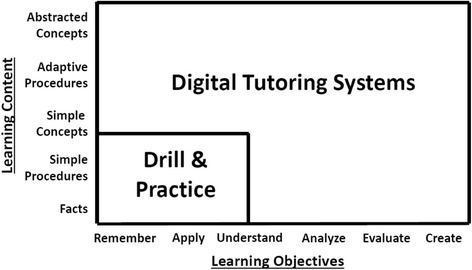
Overview of learning objectives. Suggested Roles for Intelligent Tutoring Systems and Drill & Practice (adapted from Anderson and Krathwohl 2001)

A cost-effective approach for developing the rudimentary knowledge and skills needed for STEM subjects is drill and practice (Fletcher et al. 1990). Drill and practice is an effective, and when well done, incentivizing approach. Its promise was early demonstrated for instruction concerning introductory basics, as found for beginning reading and elementary mathematics (Atkinson 1968; Atkinson and Fletcher 1972; Suppes 1964; Suppes and Morningstar 1972). The pairing of drill and practice with ITS is suggested by Fig. 1, which was adapted from Anderson and Krathwohl’s (2001) 2-dimensional elaboration of Bloom’s original taxonomy (Bloom et al. 1956). This suggestion is becoming less heretical in ITS circles. For instance, it is suggested by Nye et al. (2018) in this issue as a companion to ITS—along with their sensible caution that drill and practice may be overdone by focusing entirely on solving specific problems. As a solution, research has found the value, if not necessity, of reflection by learners on both successful and unsuccessful problem solving efforts in ITS and elsewhere. Reflection, which is enabled by tutorial exchanges in ITS, reveals the abstract and generalizable concepts underlying problems presented and increases both retention and transfer of what is learned (e.g., Gott et al. 1996; Healy et al. 2014; Koedinger, Corbett, & Perfetti, 2012; Moreno and Mayer 2007).

**Fig. 2:**
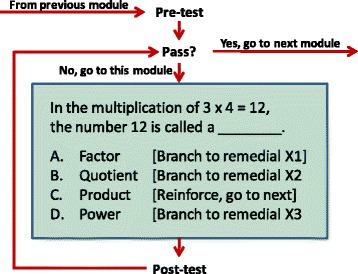
Typical programmed instruction (intrinsic programming) frame

The term “drill and practice” is occasionally viewed as an adaptation and application of programmed learning techniques to computer-assisted instruction. This approach requires instructional frames, such as the one shown in Fig. 2. These frames may be the basis for Carbonell’s 1970 definition of ITS keyed to the distinction of frame oriented versus information structure oriented design, with ITS based on the latter. In this sense, frame-oriented instruction requires the computer program to guide learners through pre-programmed material. It relies on the learner’s answers to frames such as the one shown in Fig. 2 to asses and guide progress. The burden is on human “author” of the frames to cover all states of student progress, understanding, and misunderstanding, a requirement that was found impossible to meet even for elementary school subtraction (Barr and Feigenbaum 1982). However, computer programming for frame-oriented instruction is relatively straightforward, and the approach is still widely used.

Information-structured design is more generative, flexible, and difficult to code. It requires the computer to produce more of the instructional interaction and to do so in real time. It is more flexible and resembles one-to-one dialogue with human tutors. Reviews have found frame-oriented instruction of this sort to be modestly superior to classroom learning with effect sizes around 0.35 (e.g., Kulik et al. 1982). However, results from analyses by Kulik and Fletcher (2016), VanLehn (2011), and others suggest that ITS instruction using information structures can do substantially better than that.

In the sense under consideration here, drill and practice refers to approaches developed, for instance, by Suppes for K-8 grade mathematics and Atkinson for K-3 grade beginning reading. These approaches produced effect sizes averaging about 0.80 in comparisons with classroom instruction.^1^ Drill and practice in these and similar applications generally consists of a large number of relatively discrete items, such as horizontal addition problems, spelling patterns in reading, or second language vocabulary, that can be collected or generated by computer to prepare learners for entry into a new subject matter. These items increase in difficulty as the learner passes through the material. Progress in drill and practice learning is more a matter of recognition and recall than problem solving and concept development. Nonetheless, it provides an essential foundation for the higher and more abstract levels of learning needed to develop expertise.

For example, drill and practice may march through copy, recognition, and recall as exemplified by techniques used by Fletcher and Atkinson (1972) to teach beginning reading (pre-K through grade 3). That program used Model 33 teletypewriters, headphones, and an early application of digitized audio to teach beginning reading. It was based on the assumption that most 5-year-olds have a strong command of their native language and that reading starts by matching the language in their heads with graphemes on the printed page (or screen). It began by teaching the letters of the alphabet followed by common English language spelling patterns (e.g., AT as in FAT CAT MAT). Later, the program moved to sight word vocabulary, spelling pattern anomalies (e.g., “Wind the watch in the wind”), and other beginning reading matters, thereby establishing a progression from copy, to recognition, recall, and, finally, some of the many exceptions in English orthography.

For mathematics (Pre-K through grade 7), drill and practice may begin in a similar matter, advancing from number recognition, counting, vertical addition with one column and two rows, to 7th grade operations with negative numbers as shown in Fig. 2 (Suppes and Morningstar 1972). The students enjoyed this instruction—they were on their own playing against themselves in many ways like a serious game. They came away from it with both the competencies and comfort with mathematics needed for ITS instruction in pre-algebra and algebra.

Drill and practice may be fairly sophisticated. For instance, it may use operations research models to optimize the number of items that might be mastered, given an individual learner’s rate of learning and time available, which might be a school year or a single 10-min session. Optimization of this sort for drill and practice was early discussed by a number of researchers (e.g., Chant and Atkinson 1978; Groen and Atkinson 1966; Karush and Dear 1967; Smallwood 1968; Suppes 1964). Discussion about optimization continues, but it rarely appears in practice.

Notably and as suggested here, drill and practice can provide an effective and economic prelude to the more complex, abstract, multi-factor, and multi-dimensional material that may be presented by ITS-based learning.

## ITS in training and education

Analysis to determine objectives and standards is an essential first step in the design of any system, including ITS for education and training. Both education and training are concerned with the teaching-learning process and both have much to say to each other. However, analysis may be more straightforward and readily performed for STEM training than for STEM education.

As suggested in Table 1, the differences between training and education are neither rigid nor absolute. They lie on a continuum that might be generally identified as instruction. Most training includes elements of education and most education includes elements of training. Both surgeons and Boatswain’s mates must be trained in tying knots, but both must understand when and why to use them. Both electrical engineers and electronics technicians must know how to use Ohm’s law, but both must understand when and why it must be applied—territory that appears to be best addressed by ITS.

**Table 1:** Comparison of education and training

Education	Training
Life objectives	Job/task objectives
Negotiable objectives	Fixed objectives
Cost-effectiveness	Return on investment
Includes training	Includes education

As Table 1 suggests, instruction on the education side of the instruction continuum must prepare learners for unknown futures and support the many directions an individual’s potential that education may uncover. However, instruction on the training side of the continuum must prepare learners for a known future with tasks that are relatively well understood in advance and that better lend themselves to specifiable objectives and standards for their performance.

Objectives and standards are therefore less negotiable in instruction that is closer to the training end of the instruction continuum. The well-specified and more certain objectives and standards established for training programs also lend themselves to assessment more readily than those of education programs which may require data and information from a learner’s full lifetime in determining their value. Nonetheless, ITS systems are applicable across the continuum and have been found to produce learning results in both training and education that are substantially superior to those of classroom learning.

An example provided by Skinner et al. (2018) and tending well toward the training side of the Table 1 continuum is preparing physicians to perform RALS, by using ITS. As Skinner et al. (2018) emphasize, accurate and comprehensive task analysis is particularly needed to provide interactive support during instruction and learning. Such an analysis should identify and develop capabilities needed to perform requisite tasks to standards. Cognitive task analysis may be essential in developing these capabilities.

The precision of individualization provided by ITS is a powerful asset for RALS instruction. As Skinner et al. (2018) point out, this task is complicated by the multiple, complex interactions developing in each learner’s cognition, psychomotor, and perceptual skills—a requirement that calls for the individualizing capacities of intricate one-on-one tutoring, which, of course, is expensive and difficult to schedule when done by trained and experienced human tutors. ITS can make this tutorial instruction considerably more accessible, affordable, and effective, as Skinner et al. (2018) emphasize by calling attention to the multi-modal aspects and objectives of training in this and similar areas of training. The value of ITS in these complex areas seems likely from both a monetary and performance standpoint.

Analysis to determine objectives and standards for RALS analysis training was carried out with a commendable and unusual degree of care. There are many occasions when analysis of this sort is provided solely by what the military services describe as a BOGSAT (bunch of guys sitting around a table). The RALS program provided a notable counter to such casual approaches. Skinner et al. (2018), emphasize that RALS included a strong focus on four areas: advice obtained from experts; dealing directly with the multimodal nature of the task; emphasizing consultation with acknowledged experts in the performance of the task, and covering the effects on success of emotional processes in medical teams (per Duffy et al. 2015). This process produced what Springer et al. describe as a fine-grained log of behavior fortified by a review of pedagogical best practices.

The emphasis on objectives and standards at the expert level is another important point made by Skinner et al. (2018) Most ab initio training is now expected to develop novices or journeymen under the assumption that longer time spent in training would require more time and expense. The analysis for RALS training was based on the practice of RALS experts, thereby aiming well beyond novice or, even, journeyman levels of proficiency. Springer et al. used video and think-aloud commentary by experts to develop their tutor. The aim was to accelerate the development of IT expertise without increasing training time.

Skinner et al. keyed their ITS to objectives and standards indicated by analysis of the knowledge and skill of experts, which is similar to that used to design and develop the DARPA digital tutor. Their approach may well yield results as favorable as those found for the DARPA digital tutor. Assessment of the DARPA program found that after 16 weeks (the usual time for training new ITs), Digital Tutor graduates out-scored, with effect sizes in excess of 4.0 on tests of IT knowledge and troubleshooting skill, other new sailors who graduated from a special 35-week training program in IT and other sailors with an average of 9 years of experience in the Fleet (Fletcher and Morrison 2014). In short, ITS technology may now have advanced to the point that it can substantially accelerate the acquisition of expertise without requiring additional training time.

## Mixed initiative dialogue

An expectation for the teacher-learning process in the future was articulated in the mid-1980s for guiding what became the DoD Advanced Distributed Learning Initiative (ADL) (Fletcher et al. 2007). The idea was that education and training would rely much less on classroom instruction and would primarily consist of tutorial conversations between a computer-like device^2^ and learners or workers seeking assistance to perform a task. The computer/device would not need to store locally all the data and information needed. Instead it would draw most of that, as needed and on demand, from the global information infra-sphere, which would store all of human knowledge in a form that could be downloaded to whatever device the learner had at hand (Dodds and Fletcher 2004).^3^ The basic idea, long pursued by the Department of Defense (e.g., Fletcher 2009), was to avoid the time and costs of developing (“authoring”) education and training by leaving as much of that as possible to the computer—to be assembled online and in real time.

This expectation relied one of two defining characteristics of ITS^4^ by Carbonell (1970)—a natural language, mixed-initiative dialogue conducted as a conversation between the learner (or task performer) and the technology. Carbonell pointed out that an ITS would, as in any tutorial conversation, provide mixed initiative dialogue, with questions not initiated solely by the learner or by the computer-tutor, but one in which the initiative could be assumed as needed by either. This approach is evident in Feurzeig’s (1969) Mentor program. It was applied by Brown in the late 1960s (Brown et al. 1973) and in Brown’s development of the SOPHIE system (Brown et al. 1982).

**Fig. 3:**
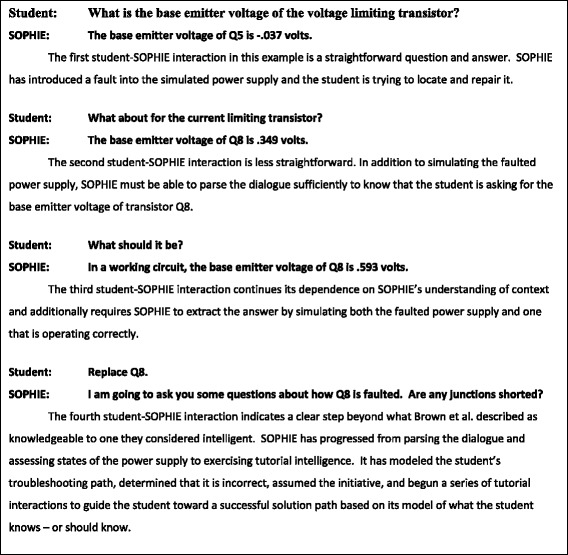
Tutorial dialogue with SOPHIE (adapted from Brown et al. 1982)

An abridged example of mixed initiative dialogue from SOPHIE is illustrated in Fig. 3. As shown by the figure, a user is learning to troubleshoot a power supply with, in this case, a fault represented by a model that SOPHIE consults. The figure shows an abridged dialogue in which the learner has the initiative and is querying SOPHIE (demonstrating SOPHIE’s impressive discourse capabilities) about the fault. This activity continues up to the point when SOPHIE, recognizing that the student is pursuing an eventually unsuccessful solution path, assumes the dialogue initiative by querying the student. Brown, Burton, & DeKleer point out that by inserting a fault into the power supply (i.e., the power supply model), being able to trace its propagation and consequences throughout the system, and responding accurately to any line of questioning initiated by the learner, SOPHIE is demonstrating knowledge. However, in knowing when to interrupt a faulty solution path and how to guide the learner to a correct path by building on what the learner already knows and/or has learned, Brown et al. concluded that SOPHIE was demonstrating intelligence.

The issues of providing guidance to learners were also addressed by Inventado et al. (2018) who provide a typical example of ITS research. Their research studied when hints should be made available in problem solving and, further, how hints should adjust to the context in which the problem is posed. Some ITS and similar systems avoid providing any direct hints because learners may simply request hints until they receive one that either directly provides a solution or becomes so focused that the answer is obvious. It is assumed that if learners start searching for hints early after an insufficient effort is made to solve a problem, very little, if anything will be learned from it. If an ITS adopts a policy of not proving hints, it instead may review what the learner already knows. Some of this review may be relevant to the learner’s current impasse and some may not. An essential learning element in these cases relies on the learner to discover what is and is not relevant for solving the problem.

Inventado et al. (2018) report data that support this approach in one experiment and contradict it in another. This contradiction remains to be resolved, but it is a good example of an ITS research issue and research being done to develop the use of ITS in STEM education and training. Their emphasis suggesting the importance of problem context (e.g., prior knowledge, assignment deadline, grade level) suggests another class of adaptations that can be made in real time by an ITS in contrast to the frame oriented approach of programmed learning and other computer-assisted systems that require designers to anticipate all possible states of the student and the instructional system—a requirement that research on ITS found cannot be met (Barr and Feigenbaum 1982).

Mixed initiative dialogue in ITS appears to be common today. Much research and many dialogue samples have been collected with the computer posing problems or providing prompts, pumps, or hints, and then passing the dialogue initiative to learner, which is where the dialogue in Fig. 3 begins. Many computer responses in ITS may be better viewed as knowledgeable than intelligent in accord with Brown et al., but others, like the context informed responses discussed by Inventado et al. (2018), may well be classified as intelligent under Brown's definition.

Graesser and others at the Institute of Intelligent Systems at the University of Memphis have long studied the tutorial interactions needed for and implemented by various developments of ITS using mixed initiative dialogue Graesser (2016). Rather than start from scratch, Graesser et al. (2018) were in an excellent position to combine the relevant capabilities of 4 ITS for the design and development of the Electronix Tutor. This system is intended to provide ab initio training for Navy sailors who will use and maintain electronic circuits. The four ITS systems were combined with AutoTutor, which has been under development and refinement in support of tutorial dialogue for at least 20 years (Graesser et al. 1995; Graesser et al. 1999; Nye et al. 2014). AutoTutor is a leading dialogue system that was developed by observing and applying successful tutorial dialogue techniques used by human tutors to provide natural language tutorial dialogue in STEM subjects. The ElectronixTutor assumes most of dialogue initiative in the system by asking questions and probing for expectations and anticipated misconceptions.

Additionally, it has long been noted that one of the best ways to develop expertise in a subject is to be required to teach it. In dealing with a variety of learner impasses and misconceptions, the teacher often gains more and, notably, deeper understanding of the subject matter and the fundamental concepts on which it is based. In recognition of this effect, Electonix uses Autotutor to require and oversee an advanced learner in assuming the dialogue initiative and teach a third dialogue agent with assistance from the computer tutor as necessary. Like SOPHIE, AutoTutor must not only know when to step in but how to rescue the student/tutor from confusing or misdirected situations.

## Discussion

Some common themes are suggested by these reflections and more importantly by the four foundational articles that were contributed to this issue by a remarkably wide collection of experienced ITS researchers and developers. These themes might include the following:

- There is an abiding need for individualization in all learning, including education and training in STEM subjects.
- STEM education and training are natural and already widely used applications for ITS, well deserving of continued attention and development.
- ITS offers an affordable means to provide individualization. But subject matter rudiments may also be provided by drill and practice, which may be a more cost-effective approach for these items. An argument can be made for pairing drill and practice techniques with ITS so that each is used to best advantage in education and training.
- Analysis to determine objectives and standards for learning is as critical for development of ITS in STEM topics as it is elsewhere. It deserves full and comprehensive attention—including considerations of context (Inventado et al. 2018) and non-cognitive modalities such as those involving psychomotor and perceptual activity (Skinner et al. 2018).
- Also indicated by Skinner et al. (2018) was the possibility and value of assigning objectives and standards for ITS intended to accelerate the acquisition of STEM expertise, beyond novice and journeymen levels of knowledge and skill, without increasing time in instruction.
- Natural language dialogue (Nye et al. 2018) possibly including a second computer-generated participant (Graesser et al. 2018) is a valuable and worthy capability provided by ITS for STEM instruction.
- It may be time to pursue the approach used by Graesser et al. (2018) of combining the best approaches and capabilities of various, existing ITS to design and build ITS for STEM. Doing so may well reduce the cost in time and effort of produce ITS—a goal of a number of funding programs.

## Conclusion

The transistion from research to routine practical implimentation remains a perennial problem. This seems particularly troublesome for education. There are no specialties in education that are equivalent to field engineering. Also the impact of education is less directly associated with economic reward than it is in other enterprises, even though its impact on every national economy is substantial. None the less, and as demonstrated by all four articles in this issue and elsewhere, we have learned much about the development of ITS systems and their promise for STEM. It may now be time to undertake more focused and serious efforts to move these capabilities out of the lab and into the field.

## Footnotes

1 Effect sizes were not in common use in the 1960s to 1970s, but may often be calculated from data reported in publications and technical reports.

2 A dubious suggestion at the time was that this device might be a telephone.

3 The Sharable Content Object Reference Model (SCORM) was an early undertaking of the ADL initiative.

4 The term, ITS, was not in vogue then. Carbonell just called it a new form of CAI.
